# Iron Speciation in Animal Tissues Using AC Magnetic Susceptibility Measurements: Quantification of Magnetic Nanoparticles, Ferritin, and Other Iron-Containing Species

**DOI:** 10.1021/acsabm.1c01200

**Published:** 2022-02-18

**Authors:** Yilian Fernández-Afonso, Laura Asín, Lilianne Beola, María Moros, Jesús M. de la Fuente, Raluca M. Fratila, Valeria Grazú, Lucía Gutiérrez

**Affiliations:** † Instituto de Nanociencia y Materiales de Aragón (INMA), CSIC-Universidad de Zaragoza, Zaragoza 50018, Spain; ‡ Departamento de Química Analítica, Universidad de Zaragoza, Zaragoza 50009, Spain; § Centro de Investigación Biomédica en Red de Bioingeniería, Biomateriales y Nanomedicina (CIBER-BBN), Zaragoza 50018 Spain; ⊥ Departamento de Química Orgánica, Universidad de Zaragoza, Zaragoza 50009, Spain

**Keywords:** magnetic nanoparticles, quantification, iron, ferritin, animal models, magnetic measurements

## Abstract

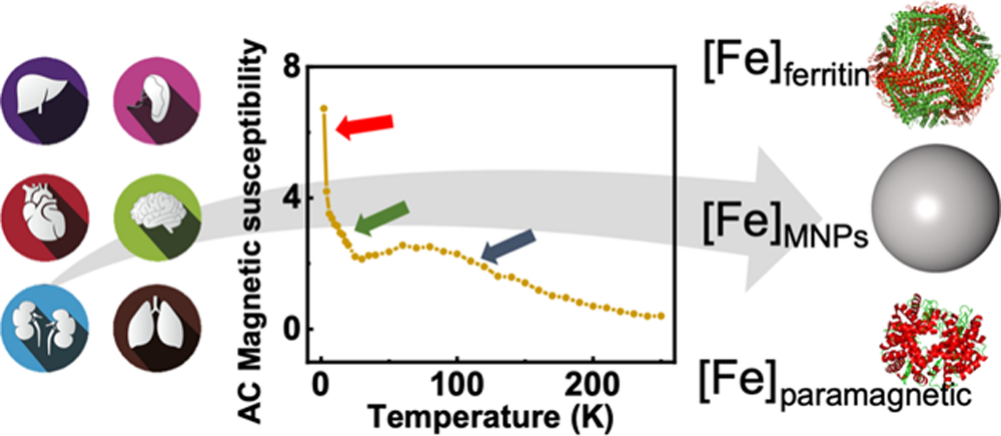

The simultaneous detection and quantification of several iron-containing species in biological matrices is a challenging issue. Especially in the frame of studies using magnetic nanoparticles for biomedical applications, no gold-standard technique has been described yet and combinations of different techniques are generally used. In this work, AC magnetic susceptibility measurements are used to analyze different organs from an animal model that received a single intratumor administration of magnetic nanoparticles. The protocol used for the quantification of iron associated with the magnetic nanoparticles is carefully described, including the description of the preparation of several calibration standard samples of nanoparticle suspensions with different degrees of dipolar interactions. The details for the quantitative analysis of other endogenous iron-containing species such as ferritin or hemoglobin are also described. Among the advantages of this technique are that tissue sample preparation is minimal and that large amounts of tissue can be characterized each time (up to hundreds of milligrams). In addition, the very high specificity of the magnetic measurements allows for tracking of the nanoparticle transformations. Furthermore, the high sensitivity of the instrumentation results in very low limits of detection for some of the iron-containing species. Therefore, the presented technique is an extremely valuable tool to track iron oxide magnetic nanoparticles in samples of biological origin.

## Introduction

Magnetic nanoparticles (MNPs), and in particular those composed of iron oxides, are extremely good candidates in the development of in vivo biomedical applications. They have already been approved for several diagnosis and treatment options, acting as contrast agents for magnetic resonance imaging (MRI), heating agents in cancer treatment by magnetic hyperthermia (MH), or iron supplements for the anemia treatment.^1^ In recent years, new biomedical approaches using these particles keep arising. Some examples include their use in wound repair,^2^ drug release from different carriers,^3^ tissue cryopreservation and rewarming,^4^ or magnetogenetics,^5^ among others.

Despite the immense potential these particles have, tracking them in vivo to study their localization, local concentration, or biodistribution is still a complicated task and hinders the fast knowledge advancement in this research area. The low amount in which these particles can be found in biological systems, sometimes on the order of nanograms or picograms, combined with the complexity of the biological matrices results in situations that resemble looking for a needle in a haystack. For example, it is easy to detect particles that have been injected intratumorally in tumor tissues, but it is difficult to discard that any of them have leaked and migrated to other organs, especially given that many commonly used techniques require significant amounts of material to produce signal above the limits of detection.

Another important difficulty is that, once in the organism, the MNP location will evolve with time. At the cellular level, nanoparticle location may change from their presence in the membrane to the incorporation in endosomes and then lysosomes, and maybe being excreted again from the cells.^6^ At the body level, the amount of injected nanomaterial that reaches a given organ is also not constant, varying over time, depending on the circulation time of the nanoparticles, their surface coating, etc.^7,8^

Furthermore, in these biological environments, the nanoparticles will transform over time. Given the ubiquitous pathways associated with iron metabolism,^9^ iron oxide magnetic nanoparticles will degrade over time, giving rise to other iron-containing species, whose nature may not be completely known.

In addition, in the case of iron oxide magnetic nanoparticles, the coexistence of endogenous species of similar composition—there is plenty of iron in our bodies—makes it complicated to use classical analytical chemistry techniques, able to determine the total amount of a given element in a tissue, as they are not able to distinguish among the endogenous species, the injected particles, or their degradation products. As a result, the study of the speciation analysis of nanomaterials in biological matrices, especially in the frame of degradation processes, is intrinsically complex.

Some of the requirements for the analysis of the presence of particles in biological systems are (i) to be able to identify the presence of particles in very low amounts, (ii) to be able to provide quantitative information regarding the presence of such particles, (iii) to be able to identify transformation processes and (iv) to be able to detect the formation of new species as a result of the transformation processes. Unfortunately, there is not a gold-standard technique widely used for this purpose.

Although there are several well-established techniques that allow the characterization of isolated particles, the truth is that not many of them are practical in complex media, because of some of the difficulties explained above. As a result, a plethora of different combinations of techniques are used for iron oxide nanoparticles characterization in biological matrices, including in vivo noninvasive techniques (such as MRI or magnetic particle imaging (MPI)) and ex vivo invasive techniques (such as optical and electronic microscopies, inductively coupled plasma-based techniques, or magnetic measurements, among many others).

In the search for alternative characterization techniques to quantify and follow the transformation of iron oxide magnetic nanoparticles in biological matrices, our group has focused on the use of a very specific type of magnetic measurement that can be performed using ex vivo tissue samples. In particular, the analysis of some contributions to the AC magnetic susceptibility of tissue samples has been used to track and quantify magnetic nanoparticles by us since 2007,^10^ verifying the effectiveness of the technique in multiple works.^11−13^ Along these years, the methodology to extract information from the magnetic characterization results has been transformed to analyze several different iron-containing species. Moreover, this methodology has been optimized to find a faster and more accurate magnetic nanoparticle quantification procedure. This work describes in detail this quantification protocol, paying attention to the limits of detection of the technique, or the recent approaches developed to be able to perform faster analysis. Furthermore, it provides the detailed description of the protocol needed for the simultaneous quantification of several iron-containing species in tissue matrices with minimal sample processing.

## Results and Discussion

Magnetic nanoparticles and tissue samples from a previously described work^14^ were used as models for the quantification analysis included in this study. In such work, 11.3 ± 1.4 nm spherical iron oxide nanoparticles coated with PMAO (poly(maleic anhydride-*alt*-1-octadecene) and functionalized with glucose were prepared.^14^ Particles were injected intratumorally to a nude mice strain that had developed a heterotopic pancreatic tumor in the right flank after the implantation of MIA PaCa-2 cells. Thirty days after the particle administration, the animals were sacrificed and the internal organs, the tumors, and the skin next to the tumors were collected and freeze-dried for their subsequent magnetic characterization.

The main objective of this work was to identify, quantify, and follow the transformations of iron oxide magnetic nanoparticles injected to animals using AC magnetic susceptibility measurements. However, as no previous separation steps were used in this characterization protocol, it was important to consider the different contributions coming from other iron-containing species present in the tissue as well as the organic part of the biological sample.

**Figure 1:**
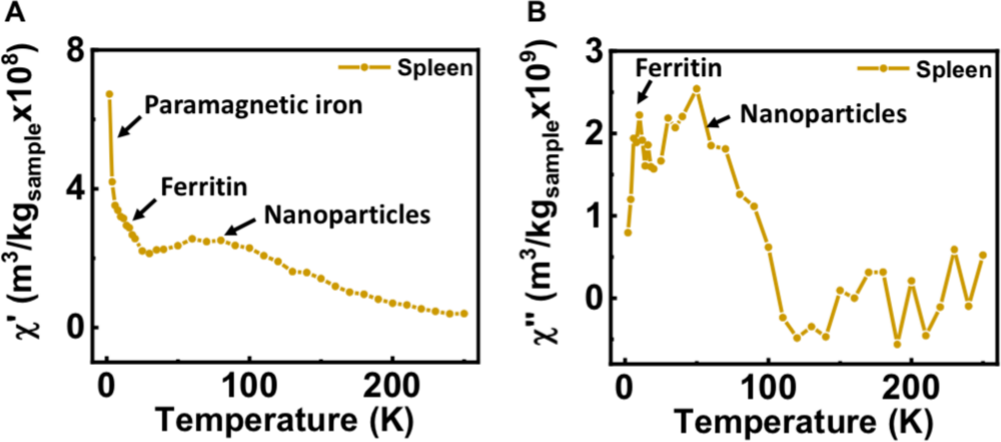
Temperature dependence of the AC magnetic susceptibility of a spleen tissue showing the contributions from the different iron-containing species. (A) In-phase component of the susceptibility, (B) out-of-phase component.

AC magnetic susceptibility measurements were performed along a wide temperature range (2–300 K in some cases) and both components of the magnetic susceptibility, the in-phase (χ′(*T*)) and the out-of-phase (χ′′(*T*)), were recorded. Several contributions to the magnetic susceptibility (see Glossary 1), originated by the presence of different iron-containing species (see Glossary 2), were found in the analysis of the different tissues (Figure 1 and Figure S1 ). A clear signal from the injected particles was easily identified in the tumors and skin samples (Figure S1).^14^ In some of the spleen tissues, such a signal was also accompanied by another one associated with the presence of ferritin (Figure 1), the iron storage protein. Furthermore, the presence of paramagnetic iron was also detected in some samples. Understanding the different magnetic contributions was fundamental for the quantification process developed.

**Figure 2:**
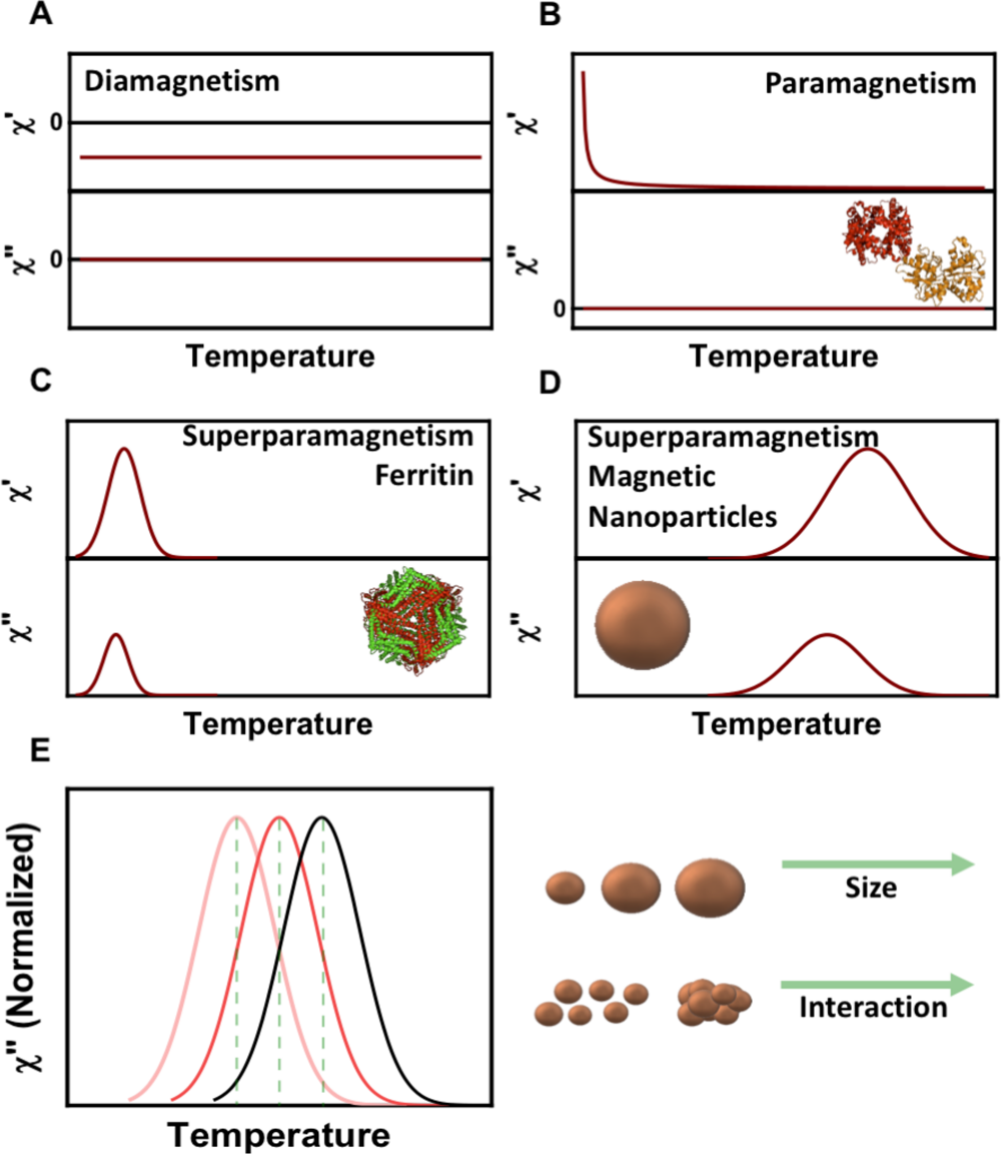
Temperature dependence of the AC magnetic susceptibility of the different iron-containing species of contributions that could be found when analyzing biological samples. (A) Diamagnetic contribution, (B) contribution from paramagnetic species, (C) contribution from ferritin iron, (D) typical contribution from magnetic nanoparticles, and (E) impact of changes in the particle size or aggregation on the temperature location of the out-of-phase susceptibility maxima from magnetic nanoparticles.

In particular, there were two distinguishable types of contributions to the magnetic susceptibility associated with iron-containing species: a paramagnetic signal and the typical signal of a relaxation process from nanoparticles (from ferritin iron and the injected particles, respectively). The paramagnetic contribution, coming probably from hemoproteins (see Glossary 1 and 2), was observed in the in-phase magnetic susceptibility of some samples (Figure 1 and Figure S1). In AC magnetic susceptibility measurements, these species presented a positive magnetic susceptibility that varied with temperature because of the influence of the thermal agitation in the alignment of the moments by the field (Figure 2).

The typical signal of iron associated with ferritin was found in some tissues that are typically associated with iron metabolism, such as the spleen (Figure 1). This signal was easily visible in the out-of-phase susceptibility in the form of a maximum at low temperatures (8–10 K) (Figures 1B and 2). This fingerprint signal was in agreement with previously described results for mouse ferritin measured in similar conditions^15^ and for ferritin from other mammals such as rats^16^ or horses.^17^ Indeed, similar signals, located at slightly lower temperatures have also been described for ferritins coming from *Xenopus laevis*(18) or *Drosophila melanogaster*.^19^

The signal arising from the particles was easily identified between 70 and 150 K in the in-phase susceptibility and 50–120 K in the out-of-phase susceptibility (Figures 1 and 2). The identification was performed by comparison with the results from the previous analysis of the injected particles.

Finally, it should also be considered that, when measuring the magnetic properties of biological samples with very low amounts of iron-containing species, a non-negligible diamagnetic contribution could be observed. This diamagnetic contribution came mainly from the organic tissue matrix (lipids, proteins, sugars, DNA, etc.) and from the sample holder (gelatin capsule), resulting in a negative contribution, of the same value in the whole temperature range, observed in the in-phase susceptibility (Figure 2).

Therefore, in this case, the tissue susceptibility was considered as a sum of different contributions described before (eq 1):
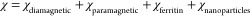
1Each of these contributions to the AC magnetic susceptibility results had its own peculiarities in order to be quantified. A protocol for the consecutive order for the determination of such different contributions was therefore developed.

### Quantification Process

To develop the procedure for the different susceptibility contribution analysis, the signal that each of the different iron-containing species presented in the in-phase and out-of-phase susceptibility components was considered. Although the organic part of the tissue samples and all the iron-containing species contributed to the in-phase susceptibility signal, only the nanoparticles and ferritin presented a contribution to the out-of-phase susceptibility component. This fact was important, as it was the key aspect that defined the structure of the quantification protocol. Therefore, the quantification process was developed in two phases. First, the out-of-phase susceptibility was analyzed, in order to quantify ferritin and the magnetic nanoparticles. The results obtained from this analysis were considered for the in-phase susceptibility analysis, to analyze the paramagnetic contribution.

As mentioned before, the analysis of each of the different iron-containing species presented specific peculiarities, and these specific aspects were considered for the analysis.

### Out-of-Phase Susceptibility Analysis

As mentioned above, only ferritin and the magnetic nanoparticles display a specific maximum in the out-of-phase susceptibility, facilitating the analysis of this component of the susceptibility, as no paramagnetic or diamagnetic contributions are reflected here. Therefore, both ferritin and the MNPs were quantified from the temperature dependence of the out-of-phase susceptibility.

### Ferritin Iron Quantification

Ferritin presented a unique singularity, which is that, given the existence of a protein shell and the weaker magnetic properties of the biomineralized iron oxyhydroxide (when compared to the magnetic nanoparticles), no dipolar interactions occurred among the iron-containing cores forming part of ferritins in close contact, simplifying the possible magnetic responses to the exposure to the AC field.

The response that was recorded for ferritin samples in the past, agreed with that of the typical superparamagnetic magnetic nanoparticle assemblies.^29^ In the limit of high temperature, ferritin behavior was similar to a Curie law, while in the limit of very low temperature all the magnetic moments were blocked. For intermediate temperatures (in the range between 5 and 30 K for ferritin in our specific measurement conditions), a maximum in both the in-phase susceptibility and the out-of-phase susceptibility occurred, with the out-of-phase susceptibility maximum located at slightly lower temperatures than the in-phase one.

**Figure 3:**
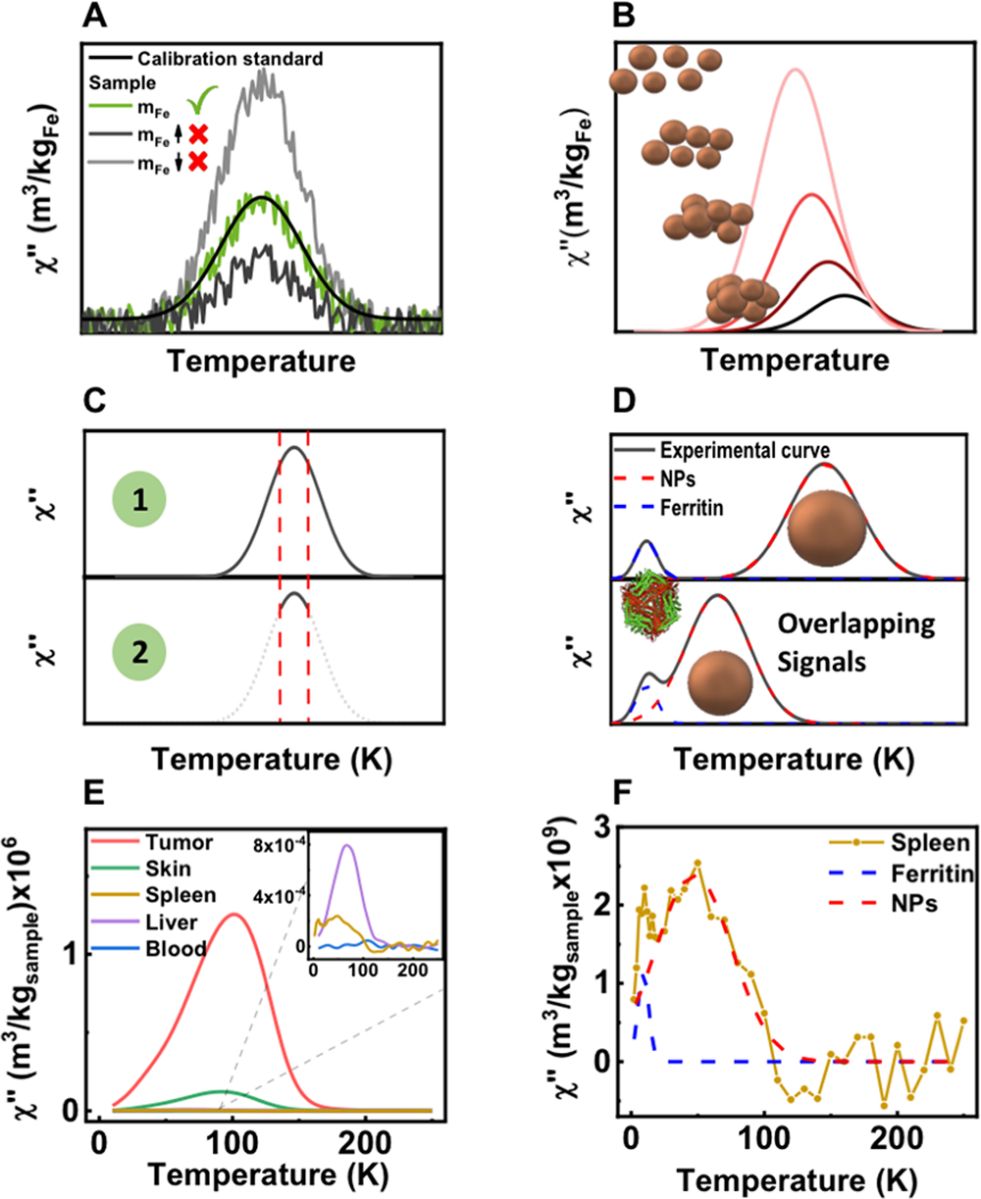
Analysis of the temperature dependence of the out-of-phase magnetic susceptibility. (A) Scheme depicting the quantification of ferritin or nanoparticles using a calibration standard. Only the correct fit allows the iron quantification. (B) Schematic representation of the magnetic behavior of MNP samples prepared with different degrees of dipolar interactions. (C) Schematic representation of the long (top) and short (bottom) measurement modes. (D) Schematic representation of the simultaneous observation of ferritin and MNPs in the out-of-phase susceptibility: (top) when both signals are independent and (bottom) when both signals are overlapped. (E) Profiles corresponding to different organs showing magnetic nanoparticles with a different degree of dipolar interactions. (F) Spleen tissue in which both ferritin and MNPs were found.

Therefore, in the case of ferritin, it was relatively straightforward to quantify the iron in the form of this species in a tissue sample. The only needed data were a “ferritin standard”, in which the iron content was known and that was previously measured in the same conditions as the tissue sample. In both samples, the tissue and the ferritin standard, the signal per iron mass associated with the ferritin out-of-phase susceptibility maxima should be the same. The height of the out-of-phase susceptibility maxima of the tissue sample was related to the ferritin standard following eq 2, and allowing the calculation of the iron content in the form of ferritin (Figure 3 A).
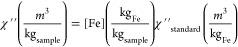
2

### Magnetic Nanoparticle Quantification

In contrast with ferritin, which is a unique species presenting a fairly reproducible behavior, magnetic nanoparticles may display a completely different response depending on their size (and size distributions), the chemical compositions and the degree of dipolar interactions (Figure 2E). The out-of-phase susceptibility profile of the nanoparticle would act as a fingerprint of the nanoparticles, but such variability would make necessary the characterization of the injected particles before their administration.

As a result, the analysis of the signal of magnetic nanoparticles in tissue samples was more complex than that of ferritin. In addition, as magnetic nanoparticles could present some degree of dipolar interactions when located in the tissue samples as a result of tissue-specific aggregation processes, specific “calibration standards” with different degrees of dipolar interactions were required. Furthermore, it had to be considered that particles could degrade over time, and deviations from the expected behavior could be due to this phenomenon.

To study the effect of dipolar interactions in the AC magnetic susceptibility signal of magnetic nanoparticles and distinguish the possible particle transformations from changes in their aggregation state, it was necessary to generate a series of dilutions of the nanoparticles where the interparticle distances were varied in a controlled manner (Figure 3B). The procedure to achieve this consisted of the generation of agar gel solutions with different amounts of magnetic nanoparticles that aimed to reach low particle concentrations that resulted in negligible interparticle interactions. This procedure was previously validated and described in further detail in previous works.^10,30−32^ In this case, the lowest mass of particles measured was formed by ∼5 μg of iron. The interesting part of this procedure was that these suspensions were prepared at temperatures slightly above ∼60 °C, at which the agar behaves as a liquid. The liquid agar solution containing the nanoparticles was then kept in a warm water ultrasonic bath. This way, the time needed for the liquid agar to go back to room temperature and become solid was extended, allowing us to have a homogeneous distribution of the particles in the agar gel.^30^ After that, this gel was freeze-dried obtaining a solid sample.

The AC magnetic susceptibility characterization of the particle samples prepared this way presented the typical signal observed for this kind of material: a single maximum in the in-phase susceptibility and a maximum in the out-of-phase susceptibility located at slightly lower temperatures. In addition, when the particle concentration in the agar sample was decreased, a shift in the temperature location of the susceptibility was observed toward lower temperatures (see scheme in Figure 3 B). This behavior was previously related to the variation in the interparticle interactions.^33−35^ In the limit for lower concentrations, the χ′′(*T*) profiles converged to a single profile. Deviations from the behavior of the most diluted “MNP calibration standards” were an indication of the interacting particle magnetic dynamics of the more concentrated MNP standards instead of the single particle dynamics of the more diluted ones. Below 0.1 wt % Fe, the interparticle interaction seemed not to affect the AC susceptibility.

For the magnetic nanoparticle iron concentration determination in tissue samples, a similar procedure as the one described for ferritin was performed (Figure 3A). The only difference now was that a magnetic nanoparticle calibration standard with a degree of dipolar interaction similar to that observed for the tissues was selected in a previous step. In fact, the optimal calibration standard was selected considering the susceptibility profile (mainly the location in temperature of the maximum and its shape), and, in some cases, the particles τ_0_ value (where τ_0_ a parameter that informs about the interparticle dipolar interactions that appears in the Arrhenius expression for the relaxation time, τ = τ_0_ exp(*E*_a_/*k*_B_*T*), where *k*_B_ is the Boltzmann constant and *E*_a_ the single particle anisotropy energy barrier. More details for the calculation can be found in Lopez et al.^10^). The height of the out-of-phase susceptibility maxima of the tissue sample was then related to the MNP selected standard following eq 2, allowing for the calculation of the iron content in the form of MNPs.

Measurements of the magnetic susceptibility as a function of temperature as the ones described in this work lasted between 3 and 5 h for each sample, depending on the number of data points recorded in the whole temperature range (≈ 5–300 K). As in many experiments involving biological samples where duplicates or triplicates involving several animals were needed, it was customary to improve the measurement protocol to be able to perform faster analysis of tissue samples, allowing the study of a multitude of organs for a quick check of the possible accumulation of particles. To do that, shorter experiments recording only AC susceptibility data over a short temperature range located around the out-of-phase susceptibility maxima were recorded (Figure 3C); for example, in the tumor, AC susceptibility data were recorded around ∼100 K (70–120 K), in a temperature range that covered the temperature position in the out-of-phase susceptibility maxima in all the tissues. This approach was fundamental to make feasible the characterization of a greater number of biological samples in a reasonable amount of time (≈ 20–30 min), also reducing the economic cost associated with the measurements.

Using the protocol described in this section, the amount of iron in the form of magnetic nanoparticles present in the tissue samples described in Figure 1 and Figure S1 was calculated to be 23 ± 3 μg in the tumor, 6.7 ± 0.8 μg in the skin, 86 ± 8 ng in the spleen, and 3.0 ± 0.3 μg in the liver (notice the very low number of particles in the spleen (3 orders of magnitude) in comparison with the rest of the organs).

### Analysis of the Out-of-Phase Susceptibility of a Sample Containing Several Iron-Containing Species

In this work, we studied the presence of particles in tumors, the skin next to tumors, the liver, the spleen, and blood (Figure 1 and Figure S1). Interestingly, the out-of-phase susceptibility signal corresponding to the particles found in all these tissues was not always the same, indicating that probably different degrees of dipolar interactions were occurring in each organ as a result of a different local aggregation in each specific organ or type of cell (Figure 3E and Figure S2). These differences suggested the need to use different MNP calibration standards for each tissue.

In some of the analyzed tissues, no signal above the background noise was detected in the out-of-phase susceptibility data. The analysis of such noise allowed us to determine the limit of detection of both ferritin and the injected particles in the tissue samples. Such a concentration was defined as the iron concentration, either in the form of ferritin or in the form of particles, that would have produced a signal three times greater than that of noise. In particular, the limits of detection for the two species were in the range of 10–20 and 0.01–0.1 μg of Fe in the form of ferritin or the injected nanoparticles, respectively. This difference of several orders of magnitude, with much higher limits of detection for ferritin than for the magnetic nanoparticles, indicated that although very small amounts of iron in the form of nanoparticles can easily be detected, a larger amount of iron in the form of ferritin is needed to get a signal, making it harder to quantify this particular endogenous iron-containing species.

From all the studied organs, only the spleens showed the signal corresponding to the presence of ferritin. In this particular case, both the ferritin and MNP maxima were slightly overlapped (see example in Figure 3D). Therefore, both contributions were considered simultaneously during the analysis of the out-of-phase susceptibility data in the quantification protocol. Finally, a verification step was performed, in which both the corresponding signal calculated for ferritin and the particles were added, to verify that the results agreed with the whole tissue profile. In this final step, some minor modifications to both the ferritin and MNP iron content were tested to obtain the optimum fit of the results. In the case of the spleen sample depicted in Figure 1, the amount of iron in the form of ferritin was determined to be 32 ± 14 μg, 3 orders of magnitude higher than the 86 ± 8 ng of iron in the form of magnetic nanoparticles mentioned above.

As a summary, the tissue out-of-phase susceptibility was simulated by adding the contributions of all the species identified at a given concentration (eq 3).
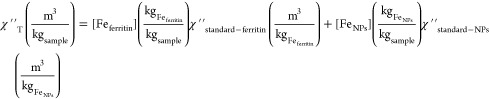
3

### In-Phase Susceptibility Analysis

Once the out-of-phase susceptibility was fully analyzed, the in-phase component of the AC magnetic susceptibility was studied. In this case, besides the contribution of the mineral species (both the ferritin biomineral cores and the injected particles), the paramagnetic and diamagnetic contributions were considered.

### Paramagnetic Iron Quantification

**Figure 4:**
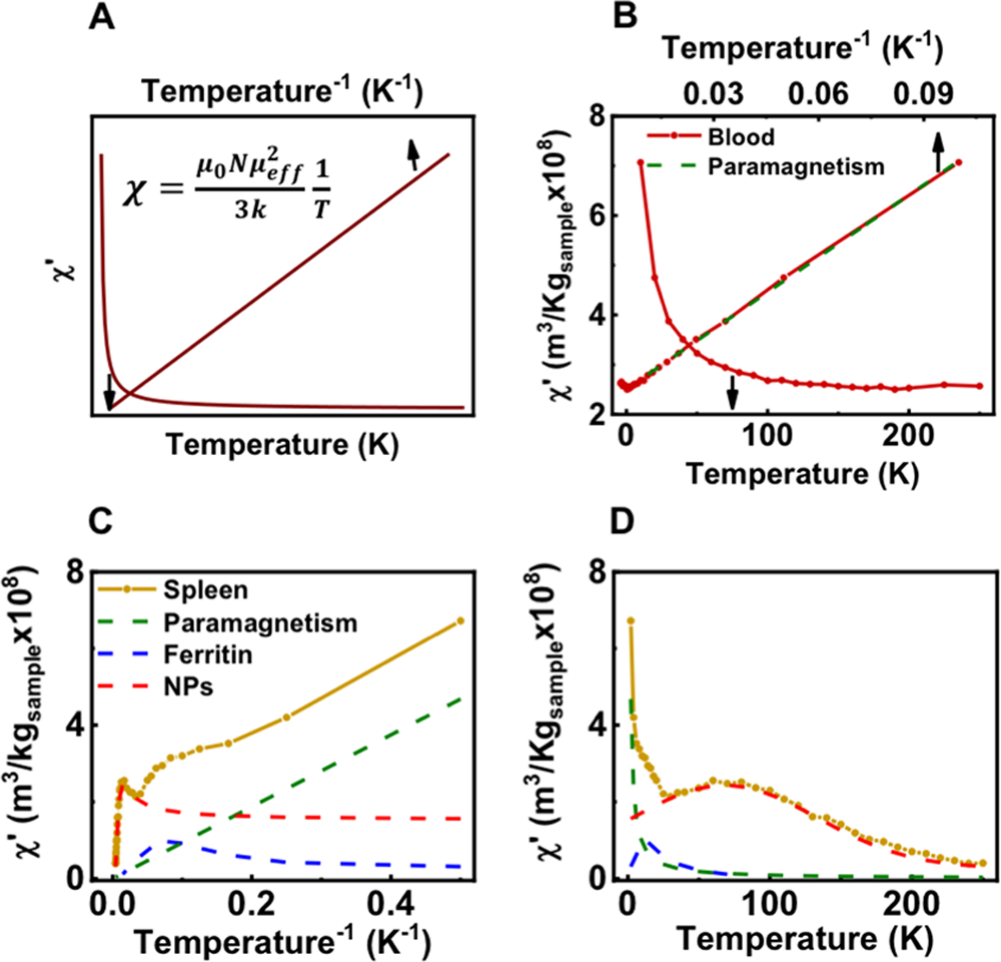
Analysis of the temperature dependence of the in-phase magnetic susceptibility of tissue samples for quantification purposes. (A) Schematic representation of the behavior of a paramagnetic species, showing the in-phase susceptibility plotted as a function of temperature and the inverse of temperature. (B) Blood sample showing the presence of paramagnetic species in the in-phase magnetic susceptibility. (C, D) Spleen sample showing the presence of several iron-containing species and the contribution calculated for each of them in the in-phase magnetic susceptibility, depicted as a function of temperature or the inverse of temperature.

The analysis of the paramagnetic contribution to the AC magnetic susceptibility presented both interesting aspects and things difficult to solve. The main difficulty was that several iron-containing species (see Glossary 2) presented a paramagnetic behavior, not allowing the distinction among these species. But, interestingly, the paramagnetic signal was easily fitted to a Curie law (eq 4 and Figure 4A). This expression described the randomizing effect of the magnetic moments’ direction that occurs when increasing the temperature and that, as a consequence, decreased the susceptibility.^36^
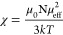
4In this equation, *T* was the temperature, μ_0_ was the magnetic permeability in a vacuum, *N* was the number of atoms per mass of iron, *k* was the Boltzmann constant, and *μ*_eff_ was the effective magnetic moment per iron ion. As a reference, in the past, it was determined that the values of the effective moment per iron ion in paramagnetic substances were around 5.5 and 5.8 μ_B_ for Fe^2+^ and Fe^3+^, respectively.^37^ Moreover, the effective moment per iron mass of deoxyhemoglobin was determined to be 5.46 μ_B_.^24^

Paramagnetic species did not present any contribution to the out-of-phase susceptibility, and thus, when analyzing biological samples, paramagnetic species could only be identified at low temperatures in the in-phase magnetic susceptibility. Unfortunately, in the high temperature range, the paramagnetic susceptibility could not be distinguished from the superparamagnetic contributions of other iron-containing species such as ferritin. The best strategy to tackle these difficulties was to perform the analysis of the paramagnetic contributions from the representation of the in-phase magnetic susceptibility versus 1/*T* (Figure 4A).

In the analysis of the in-phase susceptibility data coming from biological samples from the animal model, the simplest scenario was found for blood samples in which no ferritin or magnetic nanoparticles signals were found (Figure 4B). In such case, a clear paramagnetic signal was found, related to the iron associated with hemoglobin in such sample. This finding highlighted the importance of removing the rests of blood (e.g., by perfusion) when collecting the animal organs, as rests of blood could add a paramagnetic contribution to the tissue signal.

More challenging scenarios were found when analyzing other tissue samples where several iron-containing species were found. For example, in the spleen where both ferritin and the magnetic nanoparticles were observed, the temperature range in which the paramagnetic signal was observed was shorter than in other organs, making use of the χ′ (1/*T*) representation customary for the paramagnetism analysis (Figure 4C, D). The paramagnetic iron content was calculated from the slope of such representation in the low temperature range following eq 4, resulting in 4.8 ± 0.4 mg of iron in the form of this species in the spleen sample. In these complex samples, a final verification step was also included, in which all the contributions from iron-containing species (ferritin, MNPs, and paramagnetic iron) were considered. In this verification step, it was necessary in some cases to also include a constant diamagnetic contribution to reach the perfect fit between the tissue signal and the sum of the different species contributions.

### Comparison of the Quantification Procedure with Other Techniques

The magnetic analysis of the spleen sample shown in Figure 1 resulted in the following iron amounts: 32 ± 14 μg of iron in the form of ferritin, 4.8 ± 0.4 μg of iron in the form of paramagnetic iron, and 86 ± 8 ng of iron in the form of magnetic nanoparticles. The different order of magnitude of such values resulted in a total amount of iron in the whole sample in which the contribution of the nanoparticles was almost negligible (total amount of iron = 37 ± 14 μg of iron). This value was compared with the total iron amount measured after the acid digestion of the same organs and a spectrophotometric method based on the determination of iron(III) using 4,5-dihydroxy-1,3-benzenedisulfonic acid (Tiron) to form an iron complex that absorbs light at 480 nm.^38^ The spectrophotometric analysis indicated a total amount of iron of 64 ± 2 μg, slightly higher than the value obtained from the magnetic analysis, but on the same order of magnitude. This higher value could be explained by the existence of diamagnetic iron, which is impossible to measure with the current protocol.

The strongest signal corresponding to the nanoparticles in the rest of the analyzed organs together with the highest detection limits of ferritin iron when quantified by this technique made the comparison of the total iron amount determined by spectrophotometric methods with the quantitative analysis of the iron speciation by magnetic means unreliable.

However, in the past, the iron concentration results obtained from magnetic analysis were compared with chemical analysis when analyzing more simple biological samples. In particular, this technique was used to quantify the amount of nanoparticles (from the same batch as described in this work) internalized by cells. This model allowed the comparison of the magnetic quantification data with ICP-OES (inductively coupled plasma optical emission spectroscopy) analysis of total iron content achieving an almost perfect correlation of the results.^13^ Moreover, a good agreement was achieved between the magnetic analysis and ICP-OES measurements when analyzing tissue samples from iron-overload murine models containing mainly ferritin.^11^

### Current Challenges to the Quantification Procedure

Simpler versions of this quantification procedure were used in the past for the analysis of the biodistribution of magnetic nanoparticles^10,12,13,32,39−44^ or the presence of ferritin in iron-overload animal models.^11,16,45^

The analysis of ferritin in biological samples was relatively robust and the main difficulty was in all cases to obtain a ferritin standard of the same animal species. At the same time, once that standard was characterized, the data from the ferritin standard could be generally used in several different studies, provided that the measurement conditions applied were always the same. In fact, as most of the animal studies were performed in either mouse or rats, data from these two standards^15,16^ were used for the analysis of tissue samples coming from those animal models.

However, slight differences were found between ferritins that accumulated in different organs in some cases, probably associated with the different ferritin subunit composition and the impact of that differences on the core biomineralization.^46^ Therefore, it would be interesting for future work related to the ferritin quantification using the protocol described here to generate a library of ferritins arising from different organs.

The case of the magnetic nanoparticle analysis was more complicated. At short times after the MNP administration, it was assumed that no particle degradation had occurred, and the differences in the temperature location of the out-of-phase susceptibility maxima were associated with the presence of a different degree of dipolar interactions among the particles, allowing the selection of the most appropriated nanoparticles standard. However, if the particles remained in the organism for a considerable amount of time, degradation started to occur.^8,47^ The speed of this degradation process depended on many factors, such as the particles coating.^41^ As a result, the signal coming from the particles that were partially degraded could no longer be fitted with the standards prepared with the injected particles. Furthermore, the complexity to discern the effect of a size reduction from a decrease in the dipolar interactions among particles increased significantly. This problem remains unsolved and additional techniques are needed for the analysis of degradation processes.

**Figure 5:**
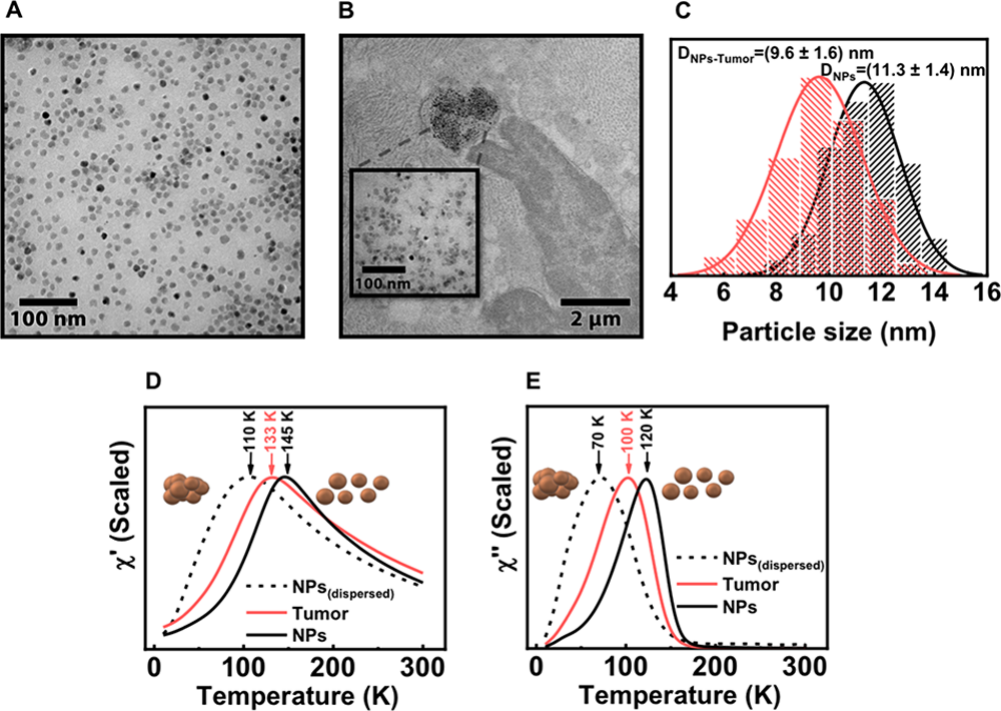
(A) Transmission electron microscopy image from the injected nanoparticles. (B) Transmission electron microscopy image of a tumor tissue extracted one month after the MNPs administration. The presence of particles is highlighted in the inset. (C) Particle size distribution corresponding to the images in A and B. (D, E) Temperature dependence of both components of the susceptibility for a tumor tissue and two MNP standards, one concentrated showing a higher degree of dipolar interaction and one dispersed showing a lower degree of dipolar interaction.

For example, in the specific case of the tissue samples described in this work, we found that, when analyzing the transmission electron microscopy images of tumor samples obtained around one month after the particle administration, a reduction in the average particle size was observed when compared to the originally injected particles in suspension (Figure 5). Particles in the tumor tissue had an average size of 9.6 ± 1.6 nm, with the whole particle size population shifted to smaller sizes than the original particles that had an average size of 11.3 ± 1.4 nm. This size reduction was not clearly detected by the magnetic characterization, as, when analyzing the tumor tissues, the out-of-phase susceptibility maxima was still found among the extreme limits of very aggregated particles and the diluted ones. In this case, several magnetic nanoparticles standards were used for the obtention of semiquantitative information regarding the amount of iron associated with the particles, indicating the error associated with the use to different standards for the analysis.

To improve the quantitative analysis of degrading nanoparticles in biological matrices, we envisaged that future studies could make use of theoretical simulations of the magnetic properties of partially degraded particles. In addition, the experimental simulation of degradation procedures would be required to gain knowledge on the transformations of the magnetic properties of the particles along their size reduction. Until these improvements are developed, this technique will still be valuable for the qualitative identification of transformations occurring over long periods of time.

### Critical Discussion on Applicability

The analysis of the iron speciation in complex biological matrices is still a difficult task to be performed using a single experimental technique. The protocol described in this work is a useful tool that could be employed as a standalone technique for the simultaneous analysis of iron-containing species, depending on the relative amount of such species. Nevertheless, we consider that a discussion of the advantages and disadvantages of the presented approach will be helpful for the researchers considering the use of this technique and our thoughts are described below.

The main disadvantages of the AC susceptibility measurements would be the need to use ex vivo samples, the time and cost associated with the measurements, and the relatively low availability of these type of instruments. Furthermore, and although it may seem trivial, this is a commonly used technique for magnetic materials characterization, but it remains highly unknown for researchers in the frame of biological sample analysis so probably collaborations between researchers with different expertise would be probably needed.

One of the most interesting advantages of this technique would be that tissue sample preparation is minimal and no separation or isolation procedures are needed for the simultaneous quantification of several iron-containing species. Furthermore, large amounts of tissue can be characterized each time. In general, all mice organs, except the liver, fit in the gelatin capsules used for the magnetic measurements. The advantage of using this large amount of material, when compared with other techniques such as TEM, is that representative results are easily obtained. This property, combined with the low limits of detection for some of the iron-containing species allows finding “the needle in the haystack” when analyzing tissues with low amounts of iron-containing species. Moreover, the high impact that small transformations of the particles have on their magnetic properties makes this approach an extremely sensible tool to track changes.

Balancing the negative and positive points, we consider that this technique remains a valuable tool for the analysis of the iron speciation in biological matrices.

## Conclusions

The protocol to obtain quantitative data about the iron speciation in tissue samples using AC magnetic susceptibility measurements was described. This protocol allowed the simultaneous quantification of several different iron-containing species present in tissue samples avoiding the use of any previous separation step. In particular, this protocol allowed the calculation of the amount of ferritin iron, iron in the form of magnetic nanoparticles, and paramagnetic iron.

A fundamental step of this protocol was to be able to identify the species present in the tissue and to have previous results on their magnetic behavior per mass of iron in the same measurement conditions. Moreover, in the case of having magnetic nanoparticle whose behavior may change depending on the dipolar interactions among them, such a degree of dipolar interactions should be determined and mimicked to produce the appropriate calibration standards.

The optimal sequence for the quantification of the different species was described. In addition, novel developments for the faster analysis of each sample were discussed. This technique provided results arising from the analysis of a relatively large amount of material, resulting in an interesting approach able to complement other techniques that were only able to analyze small portions of tissue samples (e.g., TEM). All these characteristics suggest that this tool would be extremely useful in the frame of biodistribution studies of magnetic nanoparticles or in the analysis of iron accumulation in the frame of overload diseases where a large number of tissues from animal models need to be characterized.

In the specific case of the analysis of magnetic nanoparticles, some challenges are still open. Degradation processes transforming the particle size significantly change their magnetic behavior. In such a scenario, qualitative information about the process can be obtained but new standards trying to mimic the degradation process, or theoretical simulations trying to generate the behavior of such new species, will be required for a quantitative analysis. Developing such powerful analysis techniques will be a fundamental step to obtaining information about the long-term fate of magnetic nanoparticles once they have entered living organisms.

## Materials and Methods

The detailed description of the nanoparticle synthesis and characterization and the mouse model generation were published before.^14^ A brief description of the most relevant methodological steps is provided below.

### Magnetic Nanoparticles Synthesis and Functionalization

MNPs were synthesized by a seed-mediated thermal decomposition method using iron(III) acetylacetonate (Fe(acac)_3_) as a precursor. The resulting oleic-acid-coated MNPs were then coated with poly(maleic anhydride-*alt*-1-octadecene (PMAO, MW 30 000–50 000 Da) to generate a hydrophilic surface. The MNPs were then functionalized with glucose and suspended in phosphate-buffered saline buffer (PBS) at pH 7.4. Finally, nanoparticles were passed through syringe filters (pore size of 0.22 μm) before animal administration.

### Calibration Standard Preparation

Mouse ferritin isolated from livers and hearts from Fe-dextran-loaded mice obtained during a previous work^15^ was used as quantification standard. MNP dilutions used as calibration standards were prepared by mixing different amounts of particles with hot agar gel solutions (1% w/v, 60 °C). The solidification of these hot agar particle liquid suspensions was performed by placing them in a warm water ultrasonic bath and allowing them to slowly cool to room temperature. This process provided a homogeneous distribution of the particles in the agar gel.^30^ The solid samples were then freeze-dried before the magnetic characterization sample preparation.

### Animal Model

Animals, male athymic nude mice (Crl:NU(NCr-*Foxn1*^*nu*^)), were acquired from Charles River Laboratory. Animal maintenance was held in the Animal facilities of the Centro de Investigaciones Biomédicas de Aragón - CIBA (Instituto Aragonés de Ciencias de la Salud (IACS)-Universidad de Zaragoza). To generate a tumor xenograft model, we gave 6-week old mice a single subcutaneous injection of human pancreatic MiaPaCa2 cells into the right flank. About 3 weeks later, animals received a single intratumor injection of MNPs (0.05 mL per tumor of a suspension of [Fe] = 3 mg_Fe_/mL). One month after the MNP administration, mice were euthanized by CO_2_ inhalation, and blood was directly extracted from the heart. The tumors, the skin next to them, the livers and the spleens were collected for the magnetic characterization. All animal experiments were conducted according to the law RD53/2013 and approved by the Ethics Committee for animal experiments from the University of Zaragoza that is an accredited animal welfare body.

### Magnetic Characterization of Tissue Samples and Calibration Standards

Magnetic characterization was performed using dried solid samples placed directly into gelatin capsules for their characterization. To obtain such solid samples, both the tissues and the calibration standards were freeze-dried until complete dehydration, at least 24 h. Magnetic susceptibility measurements were performed in a Quantum Design (USA) MPMS-XL SQUID magnetometer with an AC (alternating current) amplitude of 4.1 Oe at a frequency of 11 Hz. Full measurements were performed in the temperature range between 2 and 300 K to identify and quantify the presence of ferritin and MNPs in the tissues. Shorter measurements in a additional animals were performed in the 70–120 K temperature range, near the MNP maximum signal in out-of-phase magnetic susceptibility.

### Iron Concentration Analysis

The concentration of iron in the tissue samples was determined via a spectrophotometric method based on the determination of iron(III).^38^ The same tissues that were magnetically analyzed were digested in a two-step process by adding concentrated HNO_3_ and H_2_O_2_ and heating after each addition. Afterward, Milli-Q water was added to the solution. A fixed volume of this solution (50 μL) was then transferred to a 96-well plate. A solution containing a 5:1 ratio of KOH (4 N) and 4,5-dihydroxy-1,3-benzenedisulfonic acid (Tiron) was prepared separately and added (60 μL) to each well, along with a Na_3_PO_4_ solution (0.2 M, pH = 9.7, 100 μL), to a final volume of 210 μL. Samples were allowed to rest at room temperature for 15 min. Absorbance was measured at 480 nm on a spectrophotometer (Thermo Scientific, Multiskan GO) and results compared with a calibration curve obtained using iron(III) standard solutions of known concentrations treated in the same way.

## Glossary 1: Magnetic Behaviors

The classification of materials according to their magnetic behavior in response to magnetic fields at different temperatures includes five different groups of materials: diamagnetic, paramagnetic, ferromagnetic, ferrimagnetic, and antiferromagnetic.^20^ In addition, some nanometric materials may present a superparamagnetic behavior at given temperatures. The differences among them and their characteristic susceptibility is described below and depicted in Figure 2.

### Diamagnetism

In diamagnetic substances, atoms do not have a net magnetic moment because they have no unpaired electrons. Therefore, in absence of a magnetic field, the net magnetic moment of diamagnetic materials is zero. In contrast, when these substances are exposed to a magnetic field, the flux variation induces a moment opposed to the applied field. This response to a magnetic field, defined as their magnetic susceptibility, is negative in diamagnetic substances, as their magnetic moments orient in the opposite direction of the applied field, independently of the environmental temperature.

### Paramagnetism

In paramagnetic materials, some of the atoms or ions have unpaired electrons in partially filled orbitals resulting in a magnetic moment different from zero. In the absence of a magnetic field, such magnetic moments are randomly oriented and as consequence, the materials’ net magnetization is zero. However, when these materials are exposed to a magnetic field, the magnetic moments of the atoms align with the field direction, resulting in a positive susceptibility. The efficiency of the magnetic moments’ alignment depends on the environmental temperature. In particular, at increasing temperatures, a randomizing effect of the moments’ direction occurs.

### Ferro-, Ferri-, and Antiferromagnetism

In materials exhibiting these behaviors, some atoms or ions present magnetic moments that interact among them by electronic exchange forces. Depending on the relative orientation of the atom/ion magnetic moments (e.g., parallel or antiparallel) and the amount of them in each sublattice, a different behavior will result. In bulk ferro-, ferri-, or antiferromagnetic materials, there are regions in which the direction of the magnetic moments is the same, called magnetic domains. In nanomaterials, because of their small size, a situation in which just one domain exists is reached, giving rise to an interesting magnetic behavior called superparamagnetism**.** Materials presenting a superparamagnetic behavior present a zero net magnetic moment (in the absence of a magnetic field and at high enough temperatures). However, similar to what happens to paramagnetic materials, when superparamagnetic substances are exposed to a magnetic field, an alignment of the magnetic moments will occur, except that now the magnetic moments that are aligned are not from a single atom but from a particle containing various atoms.^21^

## Glossary 2: Relevant Endogenous Iron-Containing Species

In biological systems, iron is usually associated with different proteins (hemoproteins, iron–sulfur proteins, transferrin, ferritin, etc.) to avoid its toxicity.^9^ Nevertheless, only a few of them contain most of the iron in the organism. In general, it could be assumed that hemoglobin and ferritin are the two iron-containing proteins accounting for most of the endogenous iron in the organism in mammals. Although not relevant for the case described here, it has to be mentioned at this point that in pathological conditions, other not so well-defined iron-containing species (nontransferrin-bound-iron and hemosiderin) can also accumulate a significant amount of iron in the body.

### Hemoglobin

This protein is found in erythrocytes, where it is responsible for oxygen and CO_2_ transport. This protein is formed by four identical subunits composed of a globin molecule and a haem prosthetic group. In each subunit, there is one iron atom bound to the prosthetic group and the globin, keeping a coordination site available for oxygen-binding. Therefore, from a magnetism point of view, iron atoms in this protein are “far away” from each other and the protein magnetic properties depend on the iron chemical coordination (e.g., oxy- or deoxyhemoglobin depending on whether there is oxygen bound to iron). As a consequence, deoxyhemoglobin displays a paramagnetic behavior, whereas oxyhemoglobin is diamagnetic.^22−24^ In addition, in some cases, such as in hematomas, the iron oxidation state may change from Fe^2+^ to Fe^3+^, forming methahemoglobin, which is also paramagnetic.^25^

### Ferritin

Ferritin is the main iron storage protein and it conserved in nearly all living organisms. In mammals, apoferritin is a hollow spherical protein composed of 24 subunits arranged in a way that an internal cavity, of around 8 nm of diameter, is available for iron storage. Inside this protein structure, iron is accumulated forming a mineral phase whose size is limited by the cavity diameter. Therefore, nanocrystals are formed inside this cavity and their size and crystalline structure are key parameters for the magnetic behavior of this iron-containing protein. In general, at physiological conditions, a structure very similar to the iron oxyhydroxide ferrihydrite has been described,^26,27^ resulting in superparamagnetic behavior at room temperature.^17,28^ However, it has to be considered that the mineralization of other crystalline structures, especially in pathological conditions, and the possibility of several iron nanocrystals being formed simultaneously inside the protein shell have also been described in the past, which would alter the magnetic properties of ferritin.

## Supporting Information Available

Figure S1, AC magnetic susceptibility characterization of different organs (tumor, liver, skin, and blood); Figure S2, scaled out-of-phase magnetic susceptibility of several tissues and nanoparticle standards scaled to their maximum to compare the location in temperature of the maxima (PDF)
