# Supplementary material for: Iron Speciation in Animal Tissues Using AC Magnetic Susceptibility Measurements: Quantification of Magnetic Nanoparticles, Ferritin, and Other Iron-Containing Species
Source: ACS Appl Bio Mater. 2022 Feb 18;5(5):1879–89. doi: 10.1021/acsabm.1c01200 (PMC9115797; doi:10.1021/acsabm.1c01200)
Supplement: Supplementary file 1 — mt1c01200_si_001.pdf [file mt1c01200_si_001.pdf]

Iron speciation in animal tissues using AC magnetic susceptibility measurements: Quantification of magnetic nanoparticles, ferritin and other iron-containing species

Yilian Fernández-Afonso,[±, #] *Laura Asín,*[±, ≠] *Lilianne Beola,*[±]*, María Moros,*[±, ≠] *Jesús M. de la Fuente,*[±, ≠] *Raluca M. Fratila,*[±, , ≠, §] *Valeria Grazú,*[±, ≠] *Lucía Gutiérrez*[±,≠, #,*]

[±] *Instituto de Nanociencia y Materiales de Aragón (INMA), CSIC-Universidad de Zaragoza, Zaragoza 50018, Spain.*

[#] *Departamento de Química Analítica, Universidad de Zaragoza, 50009 Zaragoza, Spain.*

[≠] *Centro de Investigación Biomédica en Red de Bioingeniería, Biomateriales y Nanomedicina (CIBER-BBN), 50018 Spain.*

[§] *Departamento de Química Orgánica, Universidad de Zaragoza, 50009 Zaragoza, Spain.*

* L.G.: lu@unizar.es

KEYWORDS. Magnetic nanoparticles, quantification, iron, ferritin, animal models, magnetic measurements

Supporting Information

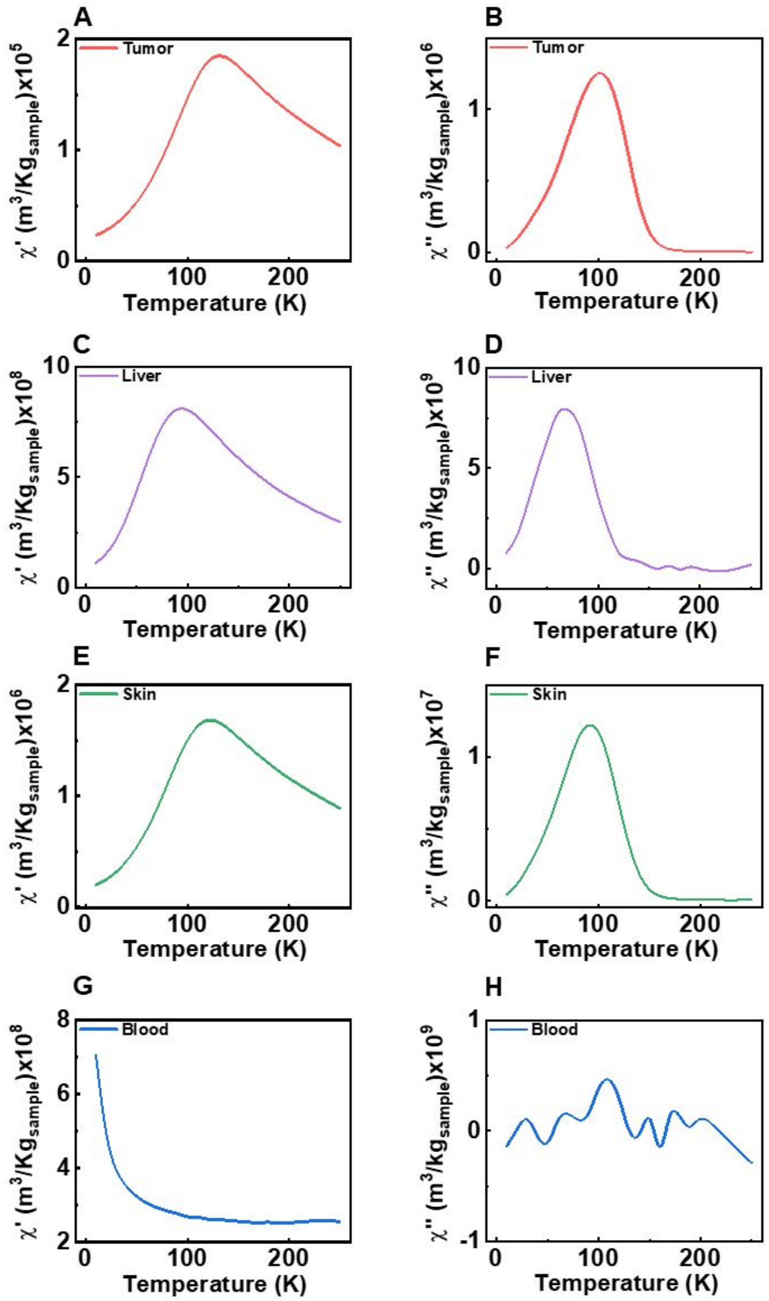

Figure S1. Temperature dependence of the AC magnetic susceptibility of several tissues showing the contributions from the different iron-containing species. A and B) Tumor, C and D) Liver, E and F) Skin, G and H) Blood.

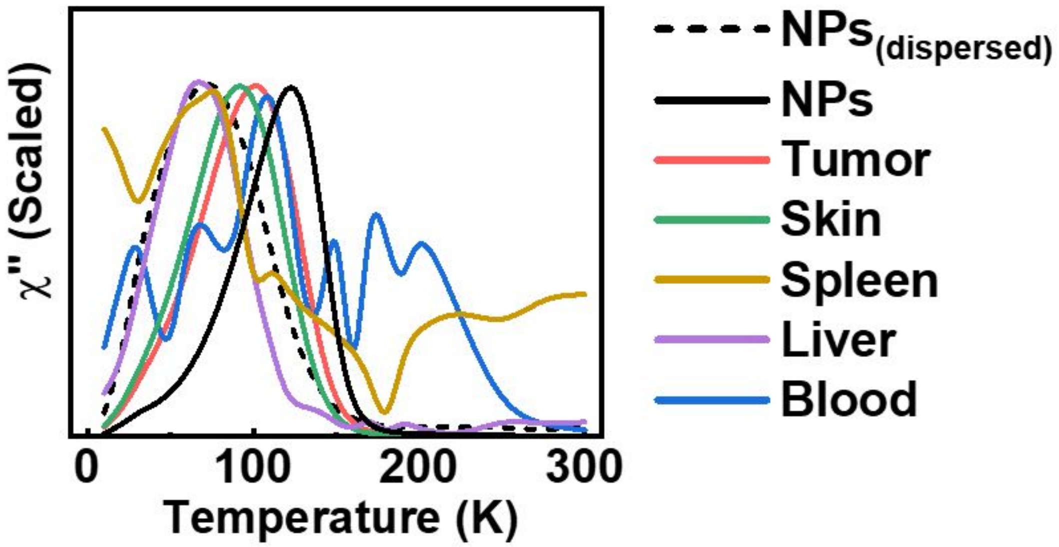

Figure S2. Temperature dependence of the out-of-phase magnetic susceptibility of two magnetic nanoparticle standards (NPs and $NPs_{(dispersed)}$ with higher and lower degree of dipolar interactions respectively) and several tissues scaled to their maxima
